# Challenges and possible solutions towards reaching global cervical cancer elimination

**DOI:** 10.1016/j.eclinm.2026.103890

**Published:** 2026-06-15

**Authors:** Laila Sara Arroyo Mühr, Joakim Dillner

**Affiliations:** Center for Cervical Cancer Elimination, F56, Karolinska Institutet and Karolinska University Hospital, 14186, Sweden

**Keywords:** Cervical cancer, Human papillomavirus, HPV vaccination, Screening, Gender neutral, Cancer elimination

## Abstract

Cervical cancer remains a leading cause of cancer-related mortality among women, despite being largely preventable through Human Papillomavirus (HPV) vaccination, HPV screening, and treatment. The World Health Organization (WHO) has called for global elimination of cervical cancer. While progress has been made in the worldwide implementation of preventive measures, the global uptake (particularly of HPV-based screening) lags behind. However, a major advance is the increased supply of HPV vaccines, propelled partly by the shift to one-dose vaccine schedules, which presents new opportunities for faster cervical cancer elimination using expanded HPV vaccination strategies such as gender-neutral vaccination, catch-up vaccination, and concomitant vaccination and screening. Another major advance is the WHO target product profile that specifies that only the 8 most important types of HPV must be screened for, which greatly improves the specificity and feasibility of HPV-based screening. This article outlines the key challenges, highlights successful examples of innovative strategies, and proposes evidence-based next steps, including specific actions on global prioritization, real-time monitoring, innovation, and equity-driven action.

## Introduction

In 2018, cervical cancer claimed the lives of over 350,000 women, despite the fact that highly effective tools for prevention are available.^1^ Human Papillomavirus (HPV) infection is the essential risk factor for the cancer and can be targeted by prophylactic vaccines that are known to not only prevent HPV-associated disease but also prevent the infection and reduce transmission of the virus in vaccinated populations. Furthermore, cervical screening using HPV testing provides a much stronger and longer lasting cancer protection than previously used screening method (cytology). Therefore WHO launched a call to action to global elimination of cervical cancer in 2018, which was followed by the 2020 WHO Global Strategy, which sets out the targets to be achieved by 2030: (1) 90% of girls fully vaccinated against HPV by the age of 15; (2) 70% of women should be screened using high-performance tests by the ages of 35 and 45; and (3) 90% of women with cervical disease should receive treatment (Table 1).^2^ While some countries have reached these goals, substantial challenges remain before global elimination of cervical cancer. In this article, we present our perspective on the key challenges and possible solutions that could accelerate progress toward cervical cancer elimination.

**Table 1:** WHO cervical cancer elimination targets.

Indicator/strategy	WHO 2030 target	Current estimate	Notes/examples
HPV vaccination coverage (≥1 dose by age 15)	90%	15–20% globally	Varies widely by country and income level
HPV-based screening (coverage at ages 35 and 45)	70%	<20%	Cytology still considered screening in some countries, with statistics not interpretable because of mixing HPV and cytology.
Treatment of identified cervical disease	90%	Limited data	Access often constrained in LMICs

## Do we have enough HPV vaccines?

While there was a shortage of HPV vaccines in the past, the global supply has greatly improved, and the world is now entering “the era of unlimited supply”. This is partly because of improved manufacturing capacity, but also because of the increasing adoption of 1- and 2-dose regimens.^3^ Historically, 3-dose schedules were recommended, but the current WHO guidance supports 1-dose or 2-dose vaccination in the ages 9–20 and 2-dose schedules for women and men above age 20.^4^ The decision has been supported by emerging evidence from observational studies and large randomized trials, for example the ESCUDDO trial in Costa Rica,^5^^,^^6^ which has confirmed that single-dose vaccination induces strong and persistent immunity.

An additional contributor is an increasing number of manufacturers entering the HPV vaccine market, with several low and middle income (LMIC) countries describing ongoing work to produce generic HPV vaccines.^7^ Prioritization used to focus on optimal use of the available number of doses but has increasingly shifted towards estimations of cost-effectiveness and comparing different possible strategies with regard to the timepoint when the cancer will be eliminated. However, only a few countries have outlined clear strategies with a defined timepoint for cancer elimination.^8^

An important development for improving access and affordability has been the local production of 2-, 4-, and 9-valent HPV vaccines in countries such as China, India, and Brazil. These efforts contribute to cost reduction, greater self-sufficiency, and improved cost-effectiveness, particularly in settings where national budgets must cover vaccination costs. Given the expanded supply, increased market competition, and the emergence of biosimilar and locally produced vaccines, there is a strong case for accelerated uptake in LMIC.

To support equitable vaccine distribution, global initiatives like Gavi, the Vaccine Alliance, and the WHO continue to play a crucial role.^9^ Gavi has been instrumental in funding and facilitating the supply of HPV vaccines to low-income countries, and through partnerships with manufacturers, the cost of the vaccines has been significantly reduced. In the Americas, the PAHO Revolving Fund has also played a key role in improving vaccine uptake through pooled procurement, enabling countries in the region to access HPV vaccines at affordable prices and with consistent supply.^10^ Further reductions in HPV vaccine prices would support faster scale-up and long-term sustainability of national vaccination programs, especially in high-burden LMIC.

## What is meant by “fully vaccinated”?

The term “fully vaccinated” has caused considerable confusion for efforts to follow up the progress of the prevention of cervical cancer. As some countries chose 1-dose and other countries chose 2-dose schedules, statistics on fully vaccinated have been difficult to compare. Although the shift to single-dose HPV vaccination now appears straightforward and highly advantageous, this was not evident at the time global targets were set.

While the WHO has endorsed single-dose HPV vaccination as an effective and cost-effective strategy for individuals aged 9–20, it has not yet officially adopted “≥1-dose by age 15” as the standard global indicator for tracking HPV vaccination coverage in relation to elimination goals. This lack of a harmonized metric complicates international comparisons and program evaluation. Given the increasing global adoption of single-dose schedules and their evidence-based effectiveness, there is a strong case for advocacy to formalize “1-dose by age 15” as the standard indicator for global monitoring. Doing so would support clarity, comparability, and alignment across countries and accelerate elimination efforts by focusing on achievable and impactful targets.

## What comes after achieving 90% vaccination coverage of young girls?

Vaccination of 90% of girls by the age of 15 is the primary goal, but as an increasing number of countries are achieving this the attention is turning to strategies with expanded vaccine use that could provide faster elimination of the disease, such as gender-neutral vaccination and concomitant vaccination and screening.^11^ Including boys & young men in HPV vaccination programs is expected to accelerate the elimination of cervical cancer by reducing transmission and more rapidly achieving herd immunity.^12^ Estimates of which effective HPV vaccination coverage is needed to obtain a complete extinction of the most dangerous HPV type (HPV16) have consistently found that 90% or more coverages are needed in girls-only HPV vaccination programs, but that extinction can be obtained using coverages of around 70–80% if both genders are vaccinated.^11^^,^^12^ Herd immunity is also achieved faster (transmission chains are broken both by immune males and by immune females). Equity (avoidance of discrimination by gender) is also an important consideration.

Another critical area is catch-up vaccination for older birth cohorts who were not vaccinated in childhood. While antibody responses in adults are generally lower than in the 9–15 age group the protection against infection and cervical disease among adult women testing HPV negative at vaccination is comparable to the protection seen among young girls.^13^

**Fig. 1:**
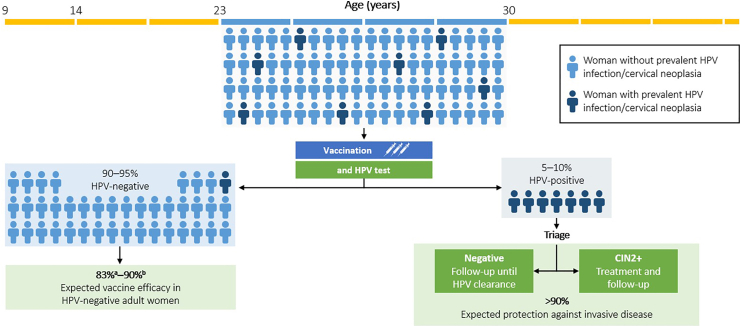
The HPV-FASTER core concept and the rationale for combined HPV screening and vaccination of adult women.

In regions where vaccine coverage is high among young girls, expanding vaccination to older age groups is an option to consider for closing the gap and ensure continued progress toward cervical cancer elimination. A particularly appealing strategy is the vaccination of young women in the age groups targeted by HPV screening programs. As most HPV infections are transient, HPV screening programs have problems with specificity (too many women testing positive who do not have any lesion) resulting in the need for triaging strategies and excessive resource consumption. However, by including HPV vaccination before screening, only older, persistent infections will be left thus improving the specificity of screening. This so-called faster strategy has the additional advantages that HPV transmission in the population is further reduced and that the protective effect of a negative screening test should last longer (HPV negativity will last longer when there are fewer new infections) (Fig. 1).^14^ This “HPV-FASTER” approach is evaluated in implementation trials in Sweden and in several LMICs.^11^^,^^15^^,^^16^

The HPV-FASTER concept promotes extending vaccination beyond adolescence into the adult screening age groups. The rationale is that vaccinating at the time of first HPV screening can reduce the prevalence of new infections, improve screening specificity (by leaving mostly persistent infections), and accelerate cervical cancer elimination. The timeline illustrates a hypothetical progression of HPV vaccination and screening activities across different age groups. The bars represent approximate age intervals: childhood vaccination (ages 9–14), followed by a potential gap before adult screening and vaccination (age ranges approximated between 23 and 30), and screening at later ages. The unmarked bars indicate flexible implementation points, not fixed time intervals. Adapted and with permission from Bosch, et al.^14^

## Do we have affordable high-performance screening tests?

Another pillar of the WHO global strategy to eliminate cervical cancer is high-performance screening, specifically the use of HPV DNA testing.^17^ Women who test HPV DNA negative have an about 7-fold lower risk of cancer than women who were negative in the previously used screening method (cytology).^18^ Another advantage of HPV-based screening is that it enables use of self-sampling, a strategy that greatly facilitates logistics and reduces costs. Self-sampling for HPV testing is equally accurate as clinician-collected samples and is highly acceptable to women, as demonstrated in multiple meta-analyses and national implementation programs, and provides similar protection against invasive cervical cancer as clinician-taken samples.^19^^,^^20^ In addition, self-sampling is culturally adaptable and can overcome many of the social, cultural, and logistical barriers that limit participation, particularly in LMICs. HPV-based screening was recommended by WHO as the preferred strategy already in 2014 but implementation has been very slow.^17^^,^^21^ The reasons for the slow implementation are not clear, but may relate to lingering perceptions that the older screening method cytology is less expensive and still affords protection.

Screening is also currently less equitably available than HPV vaccination, largely due to structural and system-level barriers, such as limited laboratory capacity, trained personnel, and logistical constraints, rather than lack of acceptance. Recent analyses, including Palmer et al., demonstrate that socioeconomic disparities are more pronounced for HPV screening than for HPV vaccination, underscoring the need for equity-driven screening strategies.^22^ Screening carries some risk of harm, including anxiety, overtreatment with obstetrical complications, and unnecessary follow-up. Therefore, screening programs should be designed to maximize benefits and minimize harms. In addition, it should be noted that vaccination programs with consistently high coverage may allow for less intensive screening strategies in the future, as vaccinated cohorts reach screening age.

Affordable HPV screening tests will be key to equitable screening implementation in LMIC. When HPV tests are acquired through public procurement processes, such as tendering, they are typically less expensive than cytology.^23^ Also, in some settings it is preferred to locally manufacture HPV tests. Examples of this are an HPV test developed by the National HPV Reference Laboratory of Brazil for use within the Brazilian national primary HPV screening program, now undergoing clinical validation (VALGENT), or the HPV test of Ukraine that is locally manufactured in the vicinity of the war zone, at manageable costs. The quality of in-country produced HPV tests can be validated using proficiency testing by the global HPV LabNet.^24^, ^25^, ^26^ Simple and inexpensive internationally comparable strategies for validation of new HPV screening tests will be essential for the progress of cervical cancer control.^27^

An important development for cervical cancer control is the WHO target product profile (TPP) of HPV screening tests that specifies that screening for only the 8 most important HPV types (HPV16, 18, 31, 33, 45, 52, 58, and 35) is necessary.^28^ This will both greatly improve the specificity of the screening and reduce the complexity of manufacturing HPV tests. There are several HPV types that have low oncogenicity that are still included in most HPV screening tests. These types are quite common, found in up to 6% of healthy women in the population, but contribute almost nothing to cervical cancer detection.^29^ We anticipate that there will be a large number of new type-restricted HPV tests developed in the coming years and that efficient international evaluation and validation strategies may be a bottleneck for fast cervical cancer control. The Global HPV Laboratory Network (HPV LabNet) works to ameliorate this bottle neck by providing internationally coordinated validation panels, external quality assurance schemes, and proficiency testing. Recognized national HPV reference laboratories that can evaluate new, type-restricted assays could greatly facilitate identification of novel tests that meet the required performance standards.

While cytology-based screening has historically contributed to reductions in cervical cancer incidence, it is important that it is widely realized that the cancer protection afforded by cytology screening is low. In populations where HPV-based screening and cytology-based screening co-exist, data show that women screened with cytology have a similar cervical cancer risk as women who were never screened, indicating that cytology-screened women should be considered ‘underscreened’ or even unscreened.^18^ If women who have had a cytology test believe that they have been screened and refrain from taking an HPV test, the use of cytology could be harmful for cervical cancer elimination efforts. This is a common problem in some high-income countries (HICs) where cytology may still be regarded as screening. It is essential that not only health authorities but also women themselves are informed of the superior cancer protection provided by HPV-based screening. Empowering women with accurate information about test performance can generate demand for better screening. Continued use of cytology, particularly if included in the “screening coverage,” can create a false sense of protection and hinder progress toward elimination. Many LMIC countries do not have the problem with pre-existing cytology screening and may therefore advance more rapidly towards cervical cancer elimination.

**Fig. 2:**
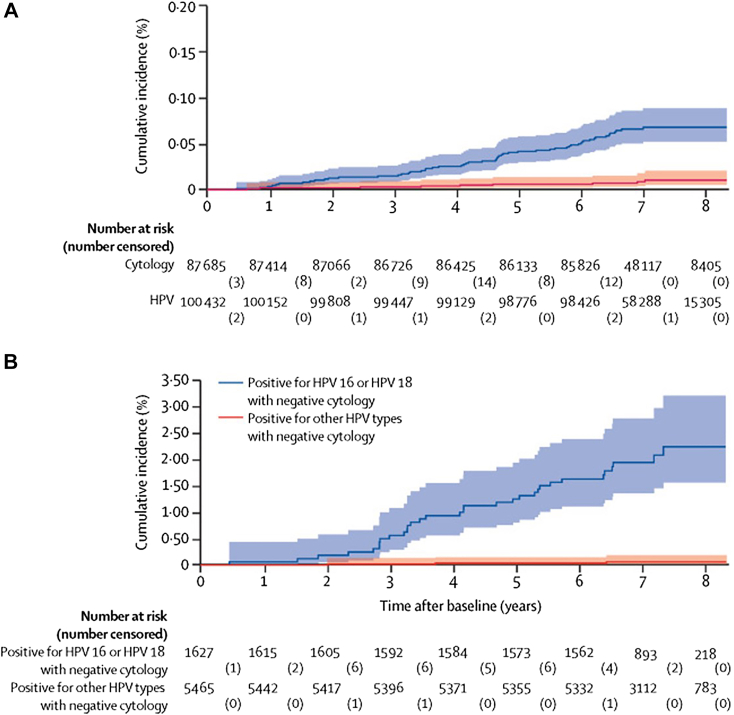
Eight-year follow-up of a large, randomized trial of HPV screening versus cytology screening. (A) Among women testing negative for HPV or cytology at baseline. (B) Among women testing positive for HPV and negative for cytology triage at baseline, by HPV genotype. Scale on the y-axis is different for (A) and (B). HPV = human papillomavirus. Adapted and with permission from Wang, et al.^18^

In a large randomized trial, the invasive cervical cancer risk among cytology-negative women was 9/100,000 and the risk among HPV-negative women was 7-fold lower (1.3/100,000) (Fig. 2, Panel A).^18^ The cancer risks were quite high among HPV-positive women who were released from follow-up because of a false negative cytology triage test, but this high risk was restricted to the 2 most oncogenic HPV types (HPV16 and HPV18) (Fig. 2, panel B).^18^ Therefore, despite the fact that there are 13 HPV types that can indeed cause cancer, focusing the screening on the most oncogenic HPV types could greatly increase feasibility at limited loss of sensitivity. Important steps for better cervical cancer control would thus be to i) insist that reporting of cervical screening coverage be restricted to HPV screening coverage and ii) mapping of which HPV screening programs that include HPV genotyping in screening.

The European Guidelines for Quality Assurance of Cervical Cancer Screening explicitly recommend against the use of cytology, which means that it is considered worse than no screening at all.^30^ Many HPV-based screening programs still rely on cytology as a triage method to determine which HPV-positive women need further diagnostic workup. Unfortunately, limitations in its performance as a triage test can compromise the benefits of HPV screening. False-negative cytology among HPV-positive women is particularly problematic for the two most oncogenic HPV types (HPV16 and 18), with such cases at high risks of developing cancer.^18^ As countries shift to HPV-based screening, the triage used is essential and alternatives to cytology such as HPV extended genotyping or molecular triage methods (e.g., methylation) should be considered.

Screening needs effective treatment of screen-positive women, third pillar of the WHO elimination strategy. Adoption of simplified treatment technologies, such as WHO-recommended thermal ablation may help in global provision of treatments.^31^

## Has cervical cancer already started to decline?

**Fig. 3:**
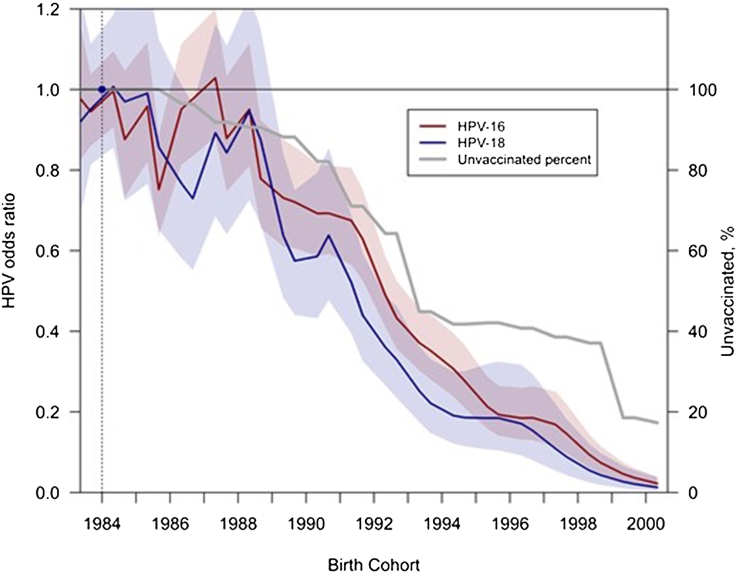
Decline in HPV16/18 Prevalence by Birth Cohort in Sweden in Relation to HPV Vaccination. The figure shows the declining prevalence of HPV types 16 and 18 among women in Sweden by birth cohort, starting with the 1984 cohort as the unvaccinated reference. The decline is plotted against the percentage of women who were not vaccinated (1 minus HPV vaccination coverage) in each cohort. The shaded area represents the 95% confidence intervals derived from an age–period–cohort model. Adapted and with permission from Gray P, et al.^32^

The earliest outcome measuring whether an HPV vaccination program is successful is if the prevalences of HPV are declining. In successful HPV vaccination programs, vaccine-targeted HPV has declined to the extent that the most dangerous HPV types (HPV16/18) have become rare (For example, Sweden found declines of 98% and 99% respectively; Fig. 3).^32^ In highly vaccinated cohorts, CIN2/3 lesions are increasingly caused by non-progressive, non-vaccine HPV types, as reported in Sweden and Finland.^33^ These types tend to have lower oncogenic potential and may not progress to cancer. This shift implies readiness for a reduced intensity screening schedule. As the prevalence of lesions due to vaccine-targeted HPV types continues to decline, programs can shift toward a “safety net” model with reduced number of lifetime screens.

The presence of lesions caused by low-oncogenic HPV types does not, on its own, justify reduced screening intensity. However, the concept of safely lowering screening intensity is supported by modeling and surveillance data from highly vaccinated populations (including Sweden, Finland, and the Netherlands),^12^^,^^32^^,^^34^ which consistently show dramatic declines in HPV16/18-related disease and markedly lower baseline risk. Furthermore, controlled trials provide additional evidence that screening intervals can be extended in vaccinated cohorts without compromising safety.^35^ Together, these findings support the rationale for exploring reduced screening frequency in settings with sustained high vaccination coverage. As the prevalence of vaccine-targeted HPV types continues to decline, programs can shift toward a “safety net” model, with reduced number of lifetime screens. Individual risk stratification, such as distinguishing between HPV types with different progressive potential, can help tailor follow-up and management, minimizing unnecessary interventions while maintaining safety.

Significant declines are reported also from moderate coverage (46–63%) programs.^34^ The benefits of vaccination are expected to take some time before vaccinated birth cohorts become older, the herd immunity is built and the gains are realized. In contrast, screening does not have a time component. In the hypothetical scenario that an entire population is screened on Monday morning and the screen-positives are managed on Monday afternoon, the full effect of the screening program is realized on Tuesday. However, the gains of implementation of HPV screening programs have so far not been as striking as expected. For example, in Sweden strong declines of cervical cancer incidences were not seen until 2023 despite the fact that HPV screening was mandated by the governmental authorities in 2015.^36^ Major reasons include i) slow and gradual implementation, where all women in the country will not have been offered HPV screening until 2028 (the last region implemented HPV screening in 2021, but as there is a 7-year screening interval all women will not have been offered HPV screening until 2028). ii) triaging of HPV positive women with cytology. A large proportion of cancers occurring in HPV screening have indeed been detected by the HPV test, but have been released by a false negative cytology test.^18^

However, rapid and radical introduction of HPV screening is feasible. For instance, Australia, Belgium and the Netherlands introduced HPV screening on a clearly defined date, with immediate, full population coverage. Thus, slow implementation is not inevitable, and rapid elimination of cervical cancer is a both feasible and desirable aim. Indeed, many countries (e.g., UK, Australia, Denmark, Sweden) report on declining prevalences of both cervical cancer precursors and of the cervical cancer itself.^37^, ^38^, ^39^, ^40^, ^41^

As the HPV prevalences keep falling, HPV-based screening will become successively easier to implement raising hopes that gains in cervical cancer incidence will be seen in the next few years.

## What are the next steps for accelerated elimination?

Although there has been very significant progress both regarding implementation of HPV vaccination, HPV screening and treatment, only a few countries will achieve elimination–defined by WHO as an age-standardized incidence of fewer than 4 cases per 100,000–within the next decade. Effective global monitoring is therefore critical to keep countries on track. A recent systematic review in eClinicalMedicine has summarized which countries are closest to meeting the WHO elimination thresholds.^22^ The review shows that Australia is on track to reach an age-standardized incidence below 4 cases per 100,000 women within the coming decade, supported by its long-standing high vaccination coverage and early adoption of HPV-based screening. A few additional countries show similar trajectories based on combined high vaccine uptake and HPV screening implementation. However, globally only a small number of countries, approximately 15, currently meet or are close to the 90% HPV vaccination target, corresponding to less than 10% of the world's female population.^22^

A most likely effective strategy would be to ensure timely availability of monitoring statistics in accordance with WHO goals, for example proportion of girls who have had at least one dose of HPV vaccine by age 15 (clearly specifying one-dose coverage as an internationally comparable reporting standard) and proportion of the population that has had an HPV test during the last 10 years at age 35 and age 45 (clearly specifying that cytology is not screening). Additions that could be considered when the primary goals have been met are e.g., the HPV vaccine coverage of boys and the coverage of catch-up vaccination campaigns (Table 2). Extension of HPV vaccination to adult age groups is included as a potential acceleration strategy, and increasing availability of polyvalent vaccines, together with the possibility of future local production, could further support cost-effective expansion of vaccination age limits (Table 2).

**Table 2:** Possible strategies to accelerate elimination of cervical cancer.

Strategy	Description	Expected impact	Notes/examples
Gender-neutral vaccination	Vaccinate boys and girls	Faster herd immunity, improved equity	Argentina, Australia, Austria, Bahamas, Bahrain, Barbados, Belgium, Belize, Bermuda, Bhutan, Brazil, British Virgin Islands, Cabo Verde, Cameroon, Chile, Colombia, Costa Rica, Czechia, Denmark, Dominica, Ecuador, El Salvador, Estonia, Finland, France, Georgia, Germany, Guatemala, Guyana, Hungary, Iceland, Ireland, Italy, Latvia, Lithuania, Luxembourg, Malta, Montenegro, New Caledonia, New Zealand, Niue, Northern Mariana Islands, Norway, Panama, Peru, Portugal, Saint Kitts and Nevis, Saint Lucia, Saint Vincent and the Grenadines, Serbia, Slovakia, Spain, Suriname, Sweden, Switzerland, Trinidad and Tobago, Turkmenistan, Turks and Caicos Islands, United Kingdom of Great Britain and Northern Ireland, Uruguay.^38^
Concomitant vaccination & screening	Combine HPV vaccination with HPV screening for young adult women	Improved screening specificity and reduced HPV transmission	Implementation trials in Sweden, Rwanda, Mexico, USA.
Catch-up vaccination	Target age groups not HPV vaccinated in childhood.	Closes immunity gaps, faster elimination	Countries currently reported by WHO as having active HPV catch-up programmes: Anguilla, Austria, Germany, Denmark, Estonia, Finland, United, Kingdom, of, Great, Britain, and, Northern, Ireland, Indonesia, Luxembourg, China, Macao, SAR, Singapore.^39^
			Other countries that have run catch-up programmes: Australia, Belgium, Bolivia, Brazil, Canada, Colombia, Denmark, France, Gambia, Germany, Guinea, Guyana, Hong Kong, Italy, Japan, Lao PDR, Norway, Peru, Philippines, Rwanda, Solomon Islands, Sweden, UK, USA, Zimbabwe
Extension of adult vaccination age range	Expanded eligibility beyond adolescence, up to 26–45 years depending on setting	Closes immunity gaps and accelerates elimination	Countries with adult vaccination and mention of 9-valent/local production
Women tested with cytology are considered as not screened	Invite both cytology-tested and never-tested women to HPV screening using the same algorithms	Ensures entire population receives adequate cancer protection as soon as possible	European Union guidelines.^30^
Use low-cost, high-performance HPV tests	Systems where HPV tests are purchased through competitive tenders have much lower costs of the HPV test. Large, centralized HPV screening laboratories can typically purchase HPV tests at much lower costs than small laboratories. International proficiency testing by the global HPV LabNet can ensure high performance testing also of locally produced tests	Affordable and reliable HPV testing	Inturrisi et al.^23^ Yilmaz et al.^26^
Screen only for the 8 highest risk HPV types in the WHO TPP	Restrict HPV testing to 16/18/31/33/35/45/52/58	Reduces false positives and need for triage (about half), minimal loss of sensitivity.	WHO TPP guidance.^28^
“Ready on Tuesday” screening campaigns	Design high-performance, rapidly deployable HPV screening programs	Faster population impact compared to low-performance program	Conceptual strategy, contrasting with previous, slow approaches
Avoid triaging where possible.	Minimize or eliminate the use of triage methods such as cytology, which are unsafe and difficult to implement in low-resource settings	Improves safety and feasibility of screening programs; reduces complexity and delays	Direct referral is justified for women with high cancer risk such as those testing HPV16/18 positive

On screening, it would be essential to monitor which countries that have increased feasibility by switching to self-sampling by the woman herself and which countries have improved specificity by using HPV genotyping for screening.

Reliable monitoring and evaluation are critical for guiding cervical cancer prevention strategies, yet many LMIC lack robust cancer registries or real-time surveillance systems. This gap hampers efforts to track progress, evaluate the effectiveness of HPV vaccination and screening programs, and allocate resources efficiently. According to IARC's Global Initiative for Cancer Registry Development, only about one-third of countries worldwide have high-quality population-based cancer registries, and the situation is particularly critical in LMICs where the cervical cancer burden is highest.^42^^,^^43^ Without functioning registries, it becomes difficult to assess incidence trends or identify populations not benefiting from current interventions. Strengthening national cancer registries and screening registries should be a priority alongside expanding access to vaccination and HPV-based screening. Innovative digital health solutions and international collaboration (e.g., through IARC, WHO, or GICR) may help fill these gaps and support real-time monitoring, especially in LMICs.

## Summary and call to action

Considering the effective preventive strategies available, the global elimination of cervical cancer is definitely an achievable goal. Although there has been significant progress, there has also been significant delays. The case of the Covid pandemic has taught us how fast progress can be made if it is urgently required. Globally, there are so many deaths from cervical cancer that an unnecessary delay of 5 years is a public health disaster comparable to the Covid pandemic. If it is prioritized to meet the challenge with continued innovation, political will, and equitable access to prevention, screening, and treatment the task is likely to succeed. Rapid access to data was instrumental in the fight against Covid and similar efforts to ensure that the world always has online data on the progress of the fight against HPV and cervical cancer are likely to be equally important.

Key next steps to take for achieving faster cervical cancer elimination after achieving the basic goals include i) expanding HPV vaccination programs to become gender-neutral and to include catch-up vaccination ii) reducing the costs of HPV screening (for example by switching to self-sampling, organization of testing and less expensive tests) iii) improving the specificity of HPV screening, avoiding the need for triaging and iv) addressing disparities in healthcare access. A collective global effort is needed to ensure that every woman and girl has access to the tools required for cervical cancer prevention, and that those who are diagnosed receive timely and effective treatment.

In LMICs cost reduction strategies, including local production of vaccines and HPV tests, inexpensive sampling using self-sampling, and simplified treatment options like thermal ablation, are desirable and essential. Many LMICs have demonstrated remarkable leadership–for example, Rwanda as an early adopter country of HPV vaccination–showing that elimination is feasible in LMICs.

When comparing the challenges for faster cervical cancer elimination between HIC and LMIC, it is noteworthy that many of the bottlenecks that impair screening progress are specific for HIC (history of low-performance screening test (cytology); slow screening implementation; expensive sampling infrastructure (healthcare personnel rather than self-sampling); over screening with poor balance benefit/harms (little attention to test specificity); expensive test validation systems) and that LMICs have a strong history of successful vaccination programs with public health impact, suggesting that if vaccines are available LMICs will prioritize to put them to good use.

A significant step forward is the recent resolution adopted at the World Health Assembly, which establishes World Cervical Cancer Elimination Day as an official day in the WHO calendar. This symbolic and strategic milestone reinforces the global commitment to elimination and aligns with the urgency of the call to action made in this paper.^44^ The time for action is now. To achieve global success, all stakeholders, including governments, international organizations, healthcare providers, and the scientific community, must commit to accelerating progress and ensuring that no one is left behind in the fight against cervical cancer.

## Contributors

Joakim Dillner conceptualised the manuscript. Laila Sara Arroyo Mühr drafted the original version of the manuscript and contributed to its conceptual development. Joakim Dillner critically revised the manuscript. Both authors approved the final version of the manuscript.

## Declaration of interests

The authors declare no competing interests.

## References

[1] Bray F., Laversanne M., Sung H. (2024). Global cancer statistics 2022: GLOBOCAN estimates of incidence and mortality worldwide for 36 cancers in 185 countries. CA Cancer J Clin.

[2] World Health Organization (2020). https://www.who.int/publications/i/item/9789240014107.

[3] Garland S.M., Bhatla N., Woo Y.L., Committee I.P. (2024). IPVS STATEMENT on HPV vaccination: no longer supply constraints: towards achieving WHO vaccine targets. Vaccine.

[4] World Health Organization (2022). https://www.who.int/publications/i/item/who-wer9750-645-672.

[5] Porras C., Sampson J.N., Herrero R. (2022). Rationale and design of a double-blind randomized non-inferiority clinical trial to evaluate one or two doses of vaccine against human papillomavirus including an epidemiologic survey to estimate vaccine efficacy: the Costa Rica ESCUDDO trial. Vaccine.

[6] One dose of HPV vaccine noninferior to two, data show. MedPage Today. https://www.medpagetoday.com/meetingcoverage/aacr/115340.

[7] Arroyo Muhr L.S., Dillner J. (2023). Biosimilar second-generation human papillomavirus vaccines. Lancet Infect Dis.

[8] Canfell K., Kim J.J., Brisson M. (2020). Mortality impact of achieving WHO cervical cancer elimination targets: a comparative modelling analysis in 78 low-income and lower-middle-income countries. Lancet.

[9] GAVI, The Vaccine Alliance Human papillomavirus vaccine support. https://www.gavi.org/types-support/vaccine-support/human-papillomavirus.

[10] Pan American Health Organization The revolving fund for access to vaccines, an engine of equity. https://www.paho.org/en/revolving-fund-access-vaccines-engine-equity.

[11] Arroyo Muhr L.S., Gini A., Yilmaz E. (2024). Concomitant human papillomavirus (HPV) vaccination and screening for elimination of HPV and cervical cancer. Nat Commun.

[12] Lehtinen M., Baussano I., Paavonen J., Vanska S., Dillner J. (2019). Eradication of human papillomavirus and elimination of HPV-related diseases - scientific basis for global public health policies. Expert Rev Vaccines.

[13] Apter D., Wheeler C.M., Paavonen J. (2015). Efficacy of human papillomavirus 16 and 18 (HPV-16/18) AS04-adjuvanted vaccine against cervical infection and precancer in young women: final event-driven analysis of the randomized, double-blind PATRICIA trial. Clin Vaccine Immunol.

[14] Bosch F.X., Robles C., Diaz M. (2016). HPV-FASTER: broadening the scope for prevention of HPV-related cancer. Nat Rev Clin Oncol.

[15] Faster elimination of HPV infection and cervical cancer using concomitant HPV vaccination and HPV screening: a demonstration Project in Rwanda. Study ID NCT06536855. NCT06536855.

[16] Leon-Maldonado L., Cabral A., Brown B. (2019). Feasibility of a combined strategy of HPV vaccination and screening in Mexico: the FASTER-Tlalpan study experience. Hum Vaccin Immunother.

[17] World Health Organization (2014). https://www.who.int/publications/i/item/9789241548953.

[18] Wang J., Elfstrom K.M., Dillner J. (2024). Human papillomavirus-based cervical screening and long-term cervical cancer risk: a randomised health-care policy trial in Sweden. Lancet Public Health.

[19] Gray P., Dillner J. (2026). RE: Interval cervical cancers after self-sampling for human papillomavirus in the general population. J Natl Cancer Inst.

[20] Martinelli M., Hassan S.S., Yilmaz E. (2025). Assessing sample adequacy and clinical performance of self-collected and clinician-collected HPV specimens using internal control Ct values. J Clin Virol.

[21] Almonte M., Hernandez M.L., Adsul P. (2024). Implementation efforts to support transition to HPV-based cervical cancer screening. Lancet Public Health.

[22] Han J., Zhang L., Chen Y. (2025). Global HPV vaccination programs and coverage rates: a systematic review. eClinicalMedicine.

[23] Inturrisi F., Berkhof J. (2022). Pricing of HPV tests in Italian tender-based settings. J Med Econ.

[24] International HPV Reference Center (2025). IHRC contributes to HPV self-sampling initiative in war-affected Ukraine. News. https://www.hpvcenter.se/2025/06/22/ihrc-contributes-to-hpv-self-sampling-initiative-in-war-affected-ukraine/.

[25] Arroyo Muhr L.S., Eklund C., Lagheden C. (2024). Continuous global improvement of human papillomavirus (HPV) genotyping services: the 2022 and 2023 HPV LabNet international proficiency studies. J Med Virol.

[26] Yilmaz E., Eklund C., Lagheden C. (2023). First international proficiency study on human papillomavirus testing in cervical cancer screening. J Clin Virol.

[27] Ramirez A.T., Clifford G.M., Dillner J. (2025). Reflections regarding validation of new HPV tests with reduced HPV genotypes: report from an IARC expert consultation. J Med Virol.

[28] World Health Organization (2024). https://www.who.int/publications/b/70232.

[29] Hortlund M., van Mol T., Van de Pol F., Bogers J., Dillner J. (2021). Human papillomavirus load and genotype analysis improves the prediction of invasive cervical cancer. Int J Cancer.

[30] European Commission Initiative on Cervical Cancer (EC-CvC) (2025). https://cancer-screening-and-care.jrc.ec.europa.eu/en/ec-cvc.

[31] World Health Organization (2021). WHO guideline for screening and treatment of cervical pre-cancer lesions for cervical cancer prevention. https://www.who.int/publications/i/item/9789240030824.

[32] Gray P., Wang J., Nordqvist Kleppe S., Elfstrom K.M., Dillner J. (2025). Population-based age-period-cohort analysis of declining human papillomavirus prevalence. J Infect Dis.

[33] Kann H., Hortlund M., Eklund C., Dillner J., Faust H. (2020). Human papillomavirus types in cervical dysplasia among young HPV-vaccinated women: population-based nested case-control study. Int J Cancer.

[34] Middeldorp M., Brouwer J.G.M., Duijster J.W. (2025). The effect of bivalent HPV vaccination against invasive cervical cancer and cervical intraepithelial neoplasia grade 3 (CIN3+) in the Netherlands: a population-based linkage study. Lancet Reg Health Eur.

[35] Ortega Llobet M., Gray P., Baussano I. (2026). Controlled trial of cervical cancer screening frequency among human-papillomavirus-vaccinated women. Int J Cancer.

[36] Elfstrom M., Gray P.G., Dillner J. (2023). Cervical cancer screening improvements with self-sampling during the COVID-19 pandemic. Elife.

[37] Falcaro M., Castanon A., Ndlela B. (2021). The effects of the national HPV vaccination programme in England, UK, on cervical cancer and grade 3 cervical intraepithelial neoplasia incidence: a register-based observational study. Lancet.

[38] Lei J., Ploner A., Elfstrom K.M. (2020). HPV vaccination and the risk of invasive cervical cancer. N Engl J Med.

[39] Lynge E., Bennekou Schroll J., Andersen B. (2024). Cervical cancer incidence in Denmark: disentangling determinants of time trend. Int J Cancer.

[40] Patel C., Brotherton J.M., Pillsbury A. (2018). The impact of 10 years of human papillomavirus (HPV) vaccination in Australia: what additional disease burden will a nonavalent vaccine prevent?. Euro Surveill.

[41] Harper D.M., Navarro-Alonso J.A., Bosch F.X. (2025). Impact of human papillomavirus vaccines in the reduction of infection, precursor lesions, and cervical cancer: a systematic literature review. Hum Vaccin Immunother.

[42] Pramesh C.S., Badwe R.A., Bhoo-Pathy N. (2022). Priorities for cancer research in low- and middle-income countries: a global perspective. Nat Med.

[43] Bray F., Znaor A., Cueva P. (2014).

[44] World Health Organization World health assembly resolution B156/CONF./13 – world cervical cancer elimination day. https://apps.who.int/gb/ebwha/pdf_files/EB156/B156_CONF13-en.pdf.

